# Deletion of *ddx4* Ovary-Specific Transcript Causes Dysfunction of Meiosis and Derepress of DNA Transposons in Zebrafish Ovaries

**DOI:** 10.3390/biology13121055

**Published:** 2024-12-16

**Authors:** Yuanyuan Chen, Xing Lin, Jing Dai, Yifan Bai, Fei Liu, Daji Luo

**Affiliations:** 1 Key Laboratory of Breeding Biotechnology and Sustainable Aquaculture, Institute of Hydrobiology, Hubei Hongshan Laboratory, Chinese Academy of Sciences, Wuhan 430072, China; chenyuanyuan@ihb.ac.cn (Y.C.); linxing@ihb.ac.cn (X.L.);; 2 College of Advanced Agricultural Sciences, University of Chinese Academy of Sciences, Beijing 100049, China; 3 Joint National Laboratory for Antibody Drug Engineering, School of Medicine, Henan University, Kaifeng 475004, China; 4 State Key Laboratory of Freshwater Ecology and Biotechnology, Institute of Hydrobiology, Chinese Academy of Sciences, Wuhan 430072, China

**Keywords:** *ddx4* (*vasa*), zebrafish, ovary, meiosis, transposon repression

## Abstract

Alternative splicing of *ddx4* (DEAD-box helicase 4), a key germline marker gene, has been reported to generate sex-specific transcripts in zebrafish gonads. The biological functions and regulatory mechanisms of the *ddx4* ovary-specific transcript (*ddx4-L*) during oogenesis remain unclear. In this study, we found that *ddx4-L* mutants, in which *ddx4-L* was specifically deleted, had enlarged ovaries but laid fewer eggs, along with having a lower fertilization rate compared to WT controls. RNA-seq analysis was performed to detect the changes in gene expression between WT and *ddx4-L* mutant ovaries. A total of 524 upregulated and 610 downregulated DEGs were identified. GO and GSEA enrichment analyses showed that genes involved in fertilization and reproduction biological processes were significantly downregulated. More specifically, we observed a remarkable reduction in Sycp1, a core component of synaptonemal complex, in *ddx4-L* mutant ovaries at both the mRNA and protein levels. In addition, the expressions of transposon elements, as well as the events of alternative splicing, alternative polyadenylation, and RNA editing, were analyzed based on the RNA-seq data. We found that the deletion of *ddx4-L* resulted in derepression of DNA transposons in zebrafish ovaries, possibly causing genome instability. In conclusion, our work demonstrates that the ovary-specific *ddx4* transcript plays important roles in oocyte meiosis and DNA transposon repression, which extends our understanding of the biological functions and regulatory mechanisms of sex-specific alternative splicing in zebrafish oogenesis and reproduction.

## Simple Summary

DEAD-box helicase 4 (*ddx4*, also known as *vasa*) is a highly conserved marker gene of germlines that plays essential roles in primordial germ cell (PGC) and gonad development. Here, we found that deletion of *ddx4-L*, the ovary-specific transcript of *ddx4* produced by alternative splicing, results in overgrowth of ovaries and reduced fertilization rates. RNA-seq analysis revealed the significant downregulation of meiosis-related gene *sycp1* and the derepress of DNA transposons in the *ddx4-L* mutant ovaries. Our work offers new insights into the biological functions of sex-specific alternative splicing in zebrafish (*Danio rerio*) oogenesis and reproduction.

## 1. Introduction

DEAD-box helicase 4 (*ddx4*), also known as *vasa*, is a highly conserved marker gene for germlines [1,2]. Ddx4 is an ATP-dependent RNA helicase that plays indispensable roles in the development of germ cells and gonads in animals. Deletion of *ddx4* orthologs in different species results in diverse defects in gonads. For instance, knockout of *ddx4* in fruit flies (*Drosophila melanogaster*) causes undifferentiated oocytes or even no oocytes in females, while males are unaffected [3,4,5]. In a *ddx4* knockout zebrafish (*Danio rerio*) model (vasa^sa6158^), all homozygotes eventually developed into sterile males only [6]. In mice (*Mus musculus*), *Ddx4* knockout leads to male infertility, while the females are completely fertile [7,8]. The species- and sex-specific functions of *ddx4* are still not fully understood.

Ddx4 protein has been reported to perform multiple biochemical functions in gametogenesis [1,2], such as regulating mRNA translation [4,9], participating in the structure and dynamics of germ granules [10,11], regulating the cell division of germ cells [3,12,13], and promoting piRNA (piwi-interacting RNA) biogenesis and transposon silencing [8,14,15]. Oogenesis is a complex and prolonged process involving the transitions of oogonia to oocyte and oocyte to egg, accompanied by a series of meiotic events [16,17]. Previous studies have shown that loss of *ddx4* causes abnormal chromosome condensation of germline stem cells and checkpoint kinase 2 (Chk2)-dependent oogenesis arrest in fruit flies [3,13]. piRNA-mediated transposon silencing is thought to be another important function of *ddx4* during oogenesis in fruit flies [14] and spermatogenesis in mice [8]. Activation of transposable elements (TEs) can cause genome instability, which is highly intolerant in germ cells and may result in defects in eggs and sterility [18]. *ddx4* has been reported to interact with piRNA processing factors such as Piwi proteins and Tudor proteins [2,8,19], and loss of *ddx4* greatly reduces the synthesis of piRNAs [8,14,15]. Nevertheless, the exact functions of *ddx4* in zebrafish oogenesis and the related regulatory mechanisms remain to be elucidated.

The sex-specific alternative splicing of *ddx4*, which generates the ovary-biased (*ddx4-L*, the long transcript of *ddx4* with exon 4) and testis-biased (*ddx4-S*, the short transcripts of *ddx4* without exon 4) transcripts in zebrafish gonads, has been reported in previous studies through RT-PCR and transcriptome analysis [20,21]. By constructing a *ddx4-L* specifically deleted zebrafish model, we have found that the ovary-biased *ddx4* isoform regulates germ granule aggregation and PGC development through a phase-separation mechanism. The reproductive defects in adult females and the underlying regulatory mechanisms have not yet been fully elucidated.

In this study, we continued to analyze the reproductive defects of the *ddx4-L* mutant zebrafish line and found that *ddx4-L* mutants had enlarged ovaries but laid fewer eggs, in addition to having reduced fertilization rates. RNA-seq analysis was performed to identify the underlying molecular alterations of RNAs in *ddx4-L* mutant ovaries. Differentially expressed genes (DEGs), as well as alternative splicing (AS) events, alternative polyadenylation (APA) events, A-to-I RNA editing events, and transposon element expression, were analyzed. We found that dysfunction of meiosis and derepress of DNA transposons might be responsible for the arrest of oogenesis in *ddx4-L* mutant ovaries and the low fertilization rate of eggs spawned by the mutants.

## 2. Materials and Methods

### 2.1. Zebrafish Maintenance

The ddx4-L knockout zebrafish line (hereinafter referred to as ddx4-E4Δ) was generated by targeting the alternative exon 4 of ddx4 [21] using the CRISPR-Cas9 technology. Wild-type (WT) and ddx4-L mutant zebrafish were maintained in a recirculating water system at 28.5 °C, under a light cycle of 14 h of day and 10 h of night, and were bred and raised according to a previous study [22]. The ddx4-L mutant line carries a 7 bp deletion in exon 4, which eliminates an MnlI restriction site. Genotyping was performed using PCR-RFLP analysis. PCR products spanning the mutation site were digested with MnlI (NEB, Ipswich, MA, USA, Cat: R0163S) and then subjected to agarose gel electrophoresis. Homozygotes exhibit an uncut band of 561 bp, and WT zebrafish exhibit two bands of 277 bp and 284 bp. Zebrafish used for experimental purposes were sacrificed by immersion in a 0.02% solution of MS222 (Sigma, St. Louis, MO, USA, cat#886-86-2) for a few minutes, until no opercular movement was observed. All experimental procedures involving zebrafish were approved by the Ethics Committee of Institute of Hydrobiology, Chinese Academy of Science (protocol code 2022-03-132).

### 2.2. RNA Extraction and RT-qPCR

After genotyping, total RNA samples were isolated from WT and ddx4-L mutant zebrafish ovaries by using the RNA isolater Total RNA Extraction Reagent (Vazyme, Nanjing, China, Cat: R401-01, China). RNA concentration was measured using the NanoDrop One (Invitrogen, Carlsbad, CA, USA). The cDNA was synthesized by using the HiScript III 1st Strand cDNA Synthesis Kit (Vazyme, Cat: R312-01, China), and qPCR was performed on the CFX96 Touch System (Bio-Rad, Hercules, CA, USA) using the 2× Taq Pro Universal SYBR qPCR Master Mix (Vazyme, Cat: Q712). The qPCR data were analyzed using the CFX Maestro 2.3 software (Bio-Rad). The 2^−ΔΔCt^ method was used with eef1a1l1 as an internal control. Primers used in this study are listed in Supplementary Table S1.

### 2.3. RNA Sequencing and Data Quality Control

RNA integrity was assessed using the RNA Nano 6000 Assay Kit on the Bioanalyzer 2100 system (Agilent Technologies, Santa Clara, CA, USA). Sequencing libraries were generated using the NEBNext^®^ UltraTM RNA Library Prep Kit for Illumina^®^ (NEB, Ipswich, MA, USA), following the manufacturer’s technical guide. The library preparations were then sequenced on an Illumina Hiseq platform, using the 2 × 150 bp paired-end configuration according to the manufacturer’s protocol. For each cDNA library, 6 G base paired-end raw reads were generated. Raw reads in fastq format were first processed by Cutadapt (v1.15) to remove adapters, reads containing poly-N, and low-quality reads. The resulting clean reads were used for all downstream analyses.

### 2.4. Differential Expression and Gene Set Enrichment Analysis

Clean reads were mapped to the zebrafish reference genome (DanRer11) using STAR (v2.6.1a), according to the user manual with the following parameters: “-runMode alignReads -outSAMtype BAM SortedByCoordinate -limitBAMsortRAM 10000000000 -readFilesCommand zcat -outFileNamePrefix -genomeDir -genomeLoad -readFilesIn”. featureCounts (v1.6.0) was adopted to count the mapped reads. Stringtie (v1.3.3b) was executed to calculate the Fragments Per Kilobase of transcript per Million mapped reads (FPKM) values of genes. Differential expression analysis was performed by DESeq2R package (v1.22.1). DEGs between WT and *ddx4-L* mutant ovaries were identified by using the threshold fold change ≥ 1 and adjusted *p*-values < 0.05. Gene Ontology (GO) enrichment analysis of DEGs was implemented using the RDAVIDWebService R package (v1.28.0). GO terms with corrected *p*-value less than 0.05 were considered significantly enriched among the DEGs. Gene set enrichment analysis (GSEA) was conducted with the clusterProfiler package (https://bioconductor.org/packages/release/bioc/html/clusterProfiler.html, accessed on 26 September 2024), with significance set at false discovery rate (FDR) ≤ 0.25 and *p* ≤ 0.05.

### 2.5. Western Blot

Zebrafish ovaries were homogenized and lysed in the RIPA Lysis Buffer (Beyotime, Cat: P0013B, Shanghai, China) containing protease inhibitors. Western blot was performed as previously described [22]. Briefly, protein lysates of ovaries were mixed with 6× loading buffer, boiled at 95–100 °C for 5 min, and stored at −20 °C prior to use. Protein samples were electrophoresed on 10% SDS-PAGE gels and transferred to PVDF membranes. The membranes were blocked in 5% skim milk dissolved in TBST buffer (20 mM Tris–HCl, 150 mM NaCl, 0.05% Tween 20, pH 7.6) for 1 h. The primary antibody solution was added and incubated overnight at 4 °C. The antibodies against zebrafish Sycp1 (customized by Frdbio, Wuhan, China) and β-tubulin (Proteintech, Cat: 66240-1-Ig, Wuhan, China) were used in this study. The membranes were washed three times in TBST for 5 min each and incubated in HRP-conjugated secondary antibodies (Cat: 111-035-003 and 115-035-003; Jackson ImmunoResearch Laboratories, West Grove, PA, USA) for 2 h. After three washes (5 min each) in TBST, the signals were detected using the Immobilon Western Chemiluminescent HRP Substrate (Merck-Millipore, Temecula, CA, USA) on the Image Quant LAS 4000 mini system (GE Health-care, Chicago, IL, USA).

### 2.6. Expression Analysis for TEs

The TE annotation files containing TE loci classifications in zebrafish genome [23] were utilized in this study. To quantify the TE expression, star2.7.10b aligner was used to map the clean reads to the GRCz11 genome with the following parameters: -runMode alignReads -outSAMtype BAM SortedByCoordinate -alignEndsType EndToEnd -outFilterMultimapNmax 10 -readFilesCommand zcat -outFileNamePrefix -genomeDir -readFilesIn -quantMode TranscriptomeSAM GeneCounts. Then, the Transcript V2.2.3 was used to count reads mapped on TEs, and DESeq2 v1.34.0 was used to perform differential expression analyses in R environment.

### 2.7. Alternative Splicing Analysis

Alternative splicing patterns were identified and calculated by rMATs python package (v4.0.2) with the following parameters: --b1, --b2, --gtf, -t, --readLength, --variable-read-length, --od, --nthread, and --tmp. The threshold of inclusion level change > 0.1 and adjusted *p*-values < 0.05. was used to identify significant differential alternative splicing events. Log-odds scores for splice sites were calculated using the maximum entropy (MaxEnt) method from the website tool (http://hollywood.mit.edu/burgelab/software.html, accessed on 26 September 2024). The start and end positions of the alternative fragments were determined using the exon component of splicing events that allows for the delineation of splice site regions across different splice types. Sequences corresponding to the splice site regions were extracted from the genome assembly (GRCz11) and subsequently employed to compute MaxEnt scores for both the 3′ splice site and 5′ splice site. The GC content and the lengths of the alternative fragments were calculated based on their designated start and end positions, using the bedtools software version 2.29.2.

### 2.8. Alternative Polyadenylation Events Analysis

BedGraph coverage files were analyzed by using Dynamic Analysis of Alternative Polyadenylation from RNA-Seq (DaPars) to perform *de novo* identification of differential 3′UTR usage between WT and *ddx4-L* mutant ovaries. The Percentage of Distal Usage Index (PDUI) score, output from DaPars, represents the percentage of distal PAS usage in the ovary samples. A higher PDUI score indicates more distal PAS site used. The PDUI scores for each gene were averaged across *ddx4-L* mutant and WT samples. The ΔPDUI was represented by 3′UTR differences. The significance of the ΔPDUI difference was assessed by the algorithm using Fisher’s exact test. Benjamini–Hochberg (BH) was used to control the false discovery rate, and 0.05 was used as a threshold to select significant hits.

### 2.9. A-to-I RNA Editing Analysis

Raw RNA-seq data were processed using an in-house developed pipeline based on the SPRINT toolkit [24]. All potential RNA editing sites were identified using the “sprint main” option within SPRINT with default parameters.

### 2.10. Statistical Analysis and Data Visualization

Statistical analyses and data visualization were performed using the R software (version 4.0.2), Python (version 3.9), and Graphpad Prism (version 9.0.0). Data are presented as mean ± SD unless otherwise noted.

## 3. Results

### 3.1. Knockout of ddx4-L Causes Immature Egg Accumulation in the Ovary and Reduced Fertilization Rate of Spawning Eggs in Zebrafish

**Figure 1:**
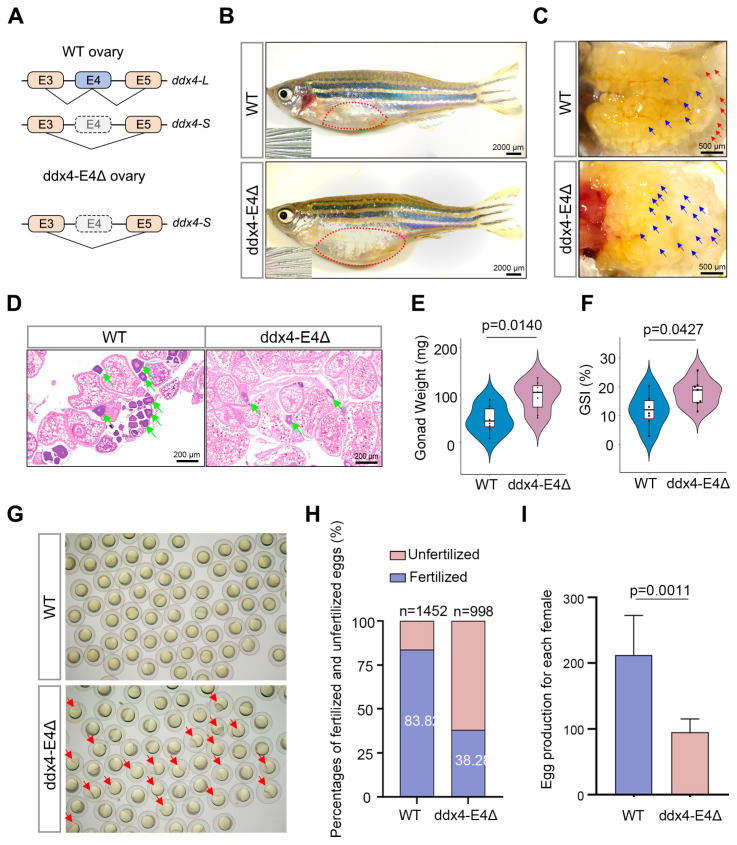
*ddx4-L* knockout causes reduced fecundity of female zebrafish. (**A**) Alternative splicing patterns of *ddx4* in WT and ddx4-E4Δ zebrafish ovaries are shown. (**B**) Abdominal morphology and secondary sexual characteristics of WT and ddx4-E4Δ zebrafish at 9 mpf. The insets show the pectoral fin of zebrafish. Red dotted frames indicate the position of ovaries. n = 7. Scale bars: 2000 μm. (**C**) Overall ovarian morphology of WT and ddx4-E4Δ zebrafish at 9 mpf. The red arrows indicate examples of mature eggs, and the blue arrows indicate examples of immature eggs. n = 7. Scale bars: 500 μm. (**D**) Hematoxylin and eosin (HE) staining of WT and ddx4-E4Δ ovarian sections at 9 mpf. The green arrows indicate early-stage oocytes, which have large, spherical nuclei stained blue or purple due to the high affinity of hematoxylin. n = 7. Scale bars: 200 μm. (**E**) Ovarian weights of WT and ddx4-E4Δ zebrafish at 9 mpf are shown. n = 7. (**F**) GSI of WT and ddx4-E4Δ females at 9 mpf are shown. n = 7. (**G**) Embryos produced by WT and ddx4-E4Δ zebrafish are shown. The arrows indicate the unfertilized embryos. (**H**) Fertilization rates of spawning eggs were quantified for WT and ddx4-E4Δ zebrafish. (**I**) The number of eggs produced by a single female during a spawning cycle was quantified. The ddx4-E4Δ females laid fewer eggs than WT controls. n = 6.

In this study, we investigated the ovarian phenotype of the *ddx4-L* mutant zebrafish abnormal oocyte differentiation by quantitative analysis of gonadal morphology, gonadosomatic index (GSI), number of spawning eggs, and fertilization rate. The schematic showed the alternative splicing patterns of *ddx4* in WT and *ddx4-L* mutant zebrafish ovaries (Figure 1A). We found that the *ddx4-L* mutant females exhibited an abnormally enlarged abdomen at 9 months post-fertilization (mpf) (Figure 1B). A significant accumulation of immature eggs, which appear to be more yellow or yellowish than mature eggs, was observed in *ddx4-L* mutant ovaries (Figure 1C). The number of early-stage oocytes was markedly reduced on the ovarian sections of *ddx4-L* mutants (Figure 1D). Body weight and weight of isolated gonad for each zebrafish individual were measured using an analytical balance. GSI was calculated by dividing gonad weight by body weight and multiplying by 100.The gonad weight and GSI were both significantly increased in *ddx4-L* mutants (Figure 1E,F and Table S2). However, the egg quality of *ddx4-L* mutants, reflected by the fertilization rate, was significantly declined (Figure 1G,H). Moreover, the number of eggs laid by *ddx4-L* mutants was also significantly lower than that of WT controls (Figure 1I and Table S2). These results suggest that the oocyte–egg transition during oogenesis may be impaired by *ddx4-L* deletion, which leads to reduced fecundity of the mutants.

### 3.2. RNA Sequencing of WT and ddx4-L Knockout Zebrafish Ovaries

**Figure 2:**
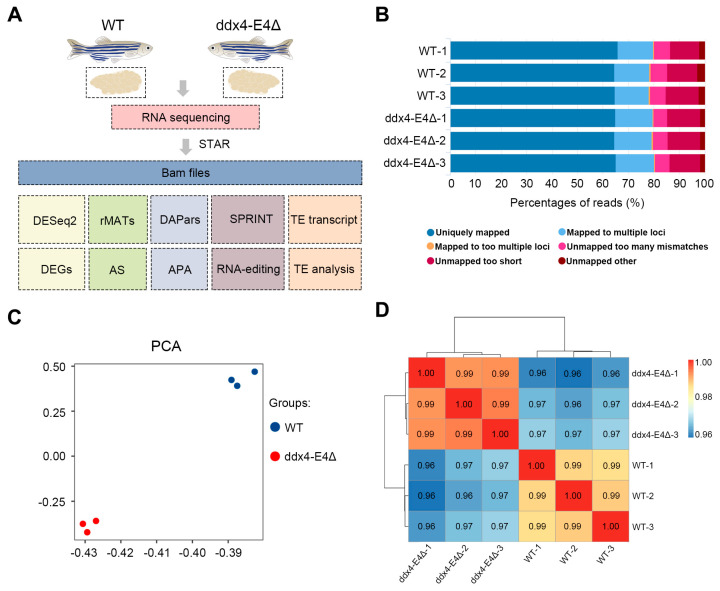
RNA sequencing of WT and ddx4-E4Δ zebrafish ovaries. (**A**) The workflow of RNA sequencing and bioinformatics analysis. Three biological replicates were employed in each experimental group. (**B**) Quality control of the RNA-seq raw data. The mapping rate of each sample is shown. (**C**) PCA analysis of WT and ddx4-E4Δ samples. (**D**) Correlation heatmap of WT and ddx4-E4Δ samples.

To explore the mechanisms underlying the defective oogenesis of *ddx4-L* mutants, we conducted RNA-seq analysis on ovaries of WT and *ddx4-L* mutants at 6 mpf. A workflow including sample collection, RNA sequencing, and bioinformatics analysis was established (Figure 2A). After filtering low-quality sequences and trimming, 20296976, 22187160, and 22091764 clean reads of WT samples; and 22089621, 21778397, 24434728 clean reads of *ddx4-L* mutant samples were obtained, respectively (Table S3). The clean reads were aligned to the zebrafish reference genome (GRCz11), and all samples yielded a mapping rate of about 80% (Figure 2B and Table S3). Principal Component Analysis (PCA) and clustering heatmap showed that WT and *ddx4-L* mutant groups had relatively small intragroup differences and relatively large intergroup differences (Figure 2C,D). These results suggest that our RNA-seq data are of high quality, and the two groups show a significant difference in global gene expression.

### 3.3. Functional Enrichment Analysis of DEGs Between WT and ddx4-L Mutant Ovaries

**Figure 3:**
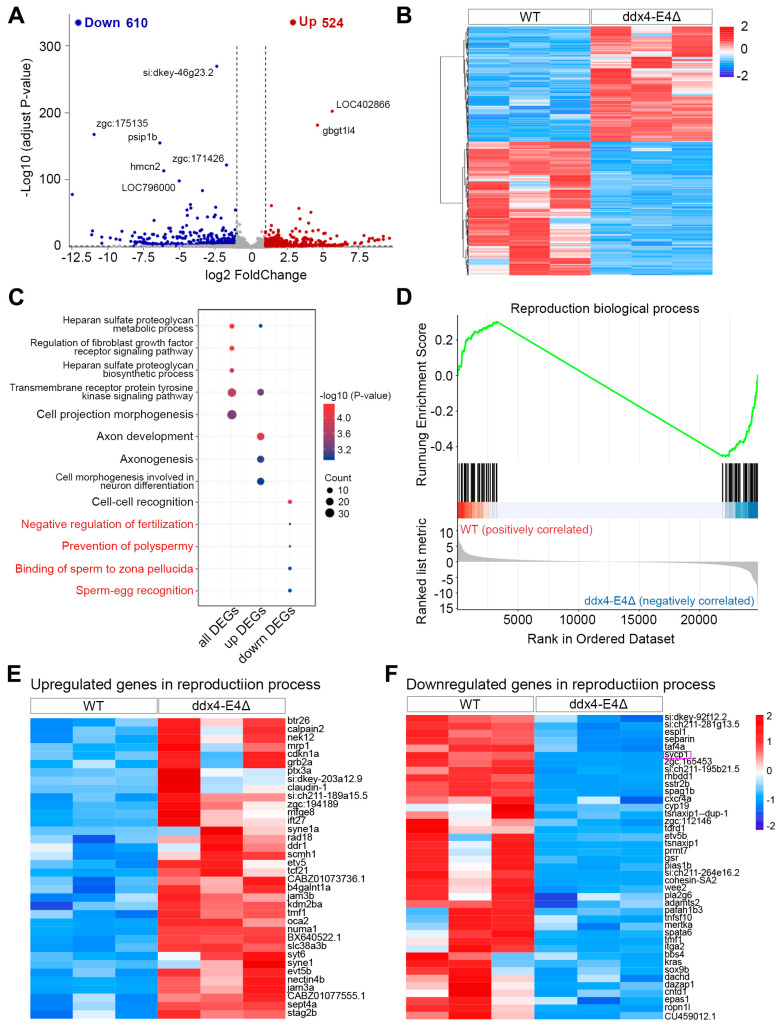
Identification and functional enrichment analysis of DEGs between WT and ddx4-E4Δ zebrafish ovaries. (**A**) The volcano plot shows the 524 upregulated and 610 downregulated genes in ddx4-E4Δ ovaries. (**B**) The heatmap shows the expression pattern of DEGs between WT and ddx4-E4Δ ovaries. (**C**) Enriched GO terms in all, upregulated, and downregulated DEGs, respectively. (**D**) GSEA analysis shows the global upregulation of genes involved in reproduction biological process. (**E**) The expression pattern of upregulated genes in the reproduction biological process term. (**F**) The expression pattern of downregulated genes in the reproduction biological process term. The purple box indicates the significantly down-regulated meiosis-related gene *sycp1*.

A total of 1134 DEGs, including 524 upregulated and 610 downregulated genes, were identified (Figure 3A and Table S4). The expression pattern of DEGs between WT and *ddx4-L* mutant ovaries are shown in the heatmap (Figure 3B). Go enrichment analysis showed that the GO terms related to the fertilization process, including negative regulation of fertilization, prevention of polyspermy, binding of sperm to zona pellucida, and sperm–egg recognition, were significantly enriched in downregulated DEGs (Figure 3C and Table S5). GSEA analysis revealed a global decrease in the expression of genes involved in the reproduction biological process in the *ddx4-L* mutant ovaries (Figure 3D and Table S5). Expression patterns of the upregulated and downregulated reproduction biological process-related genes are shown in the heatmap based on the RNA-seq data (Figure 3E,F). We found that *sycp1*, a meiosis-related gene [25], was significantly downregulated in *ddx4-L* mutant ovaries (Figure 3F). Other genes involved in reproduction biological processes, such as *tdrd1*, *cxcr4a*, *cyp19*, *wee2*, and *sox9b*, were also found to be downregulated in *ddx4-L* mutant ovaries (Figure 3F). The downregulated values of *sycp1*, *tdrd1*, *cxcr4a*, *cyp19*, *wee2*, and *sox9b* in *ddx4-L* mutant ovaries are 99%, 56%, 62%, 92%, 68%, and 67%, respectively. Sycp1 is a component of synaptonemal complex [25]. Wee2 is a kinase involved in the inhibition of oocyte meiosis [26]. Tdrd1 is a phase-separation protein involved in germ granule assembly and piRNA biogenesis [27]. Cxcr4a is a chemokine receptor playing an essential role in PGC migration [28]. Cyp19 is a rate-limiting enzyme for estrogen biosynthesis [29]. Sox9b is a transcription factor belonging to the Sry-related HMG box family and plays an important role in germ cell maintenance and ovary–testis transformation [30].

### 3.4. Deletion of ddx4-L Significantly Decreases the Expression of sycp1 During Oogenesis

**Figure 4:**
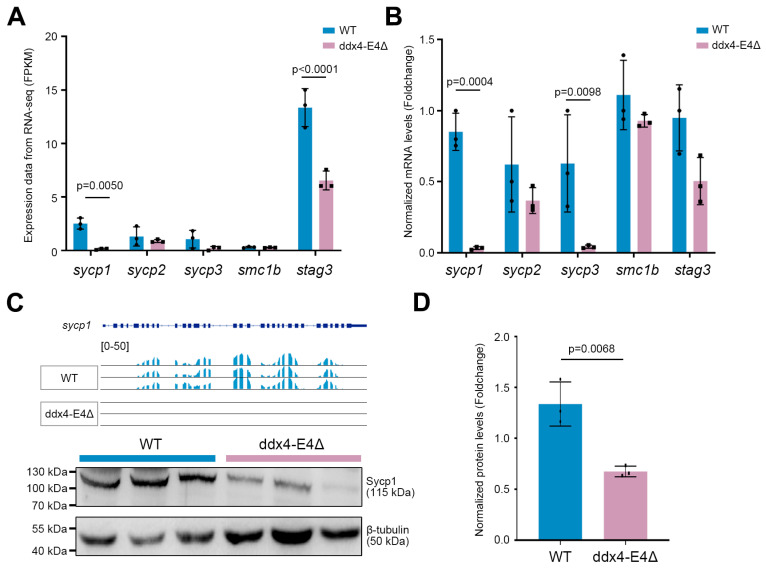
*ddx4-L* knockout leads to significant downregulation of *sycp1* at mRNA and protein levels. (**A**) Expression levels of several meiosis-related genes in WT and ddx4-E4Δ ovaries based on the RNA-seq data. The FPKM values are shown. n = 3. (**B**) qPCR validation of the mRNA levels of meiosis-related genes in WT and ddx4-E4Δ ovaries at 6 mpf. n = 3. (**C**) Detection of Sycp1 protein levels in WT and ddx4-E4Δ ovaries at 6 mpf by Western blot assay. RNA-seq tracks of *sycp1* gene in WT and ddx4-E4Δ ovaries are shown in the top panel. Original western blot images are shown in Figure S1A. (**D**) Quantitative analysis of the bands in (**C**) shows the changes in Sycp1 protein levels in ddx4-E4Δ ovaries. n = 3.

To further investigate the expression changes in meiosis-related genes in the *ddx4-L* mutant ovaries, we analyzed the mRNA levels of *sycp1*, *sycp2*, *sycp3*, *smc1b*, and *stag3* based on the RNA-seq data (Figure 4A) and qPCR data (Figure 4B). The downregulation of *sycp1*, *sycp3*, and *stag3* revealed by RNA-seq analysis was validated by qPCR results. Moreover, we generated an anti-zebrafish Sycp1 antibody according to a previous study [31] to detect the protein levels of Sycp1. We found that the Sycp1 protein was also significantly decreased in the *ddx4-L* mutant ovaries (Figure 4C,D). In considering the pivotal function of Sycp1 in meiosis and the abnormal accumulation of immature eggs in the mutant ovaries, we believe that *ddx4-L* plays an important role in oocyte meiosis by regulating the expression of meiosis-related genes.

### 3.5. Ablation of ddx4-L Causes Derepress of DNA Transposons in ddx4-L Mutant Ovaries

**Figure 5:**
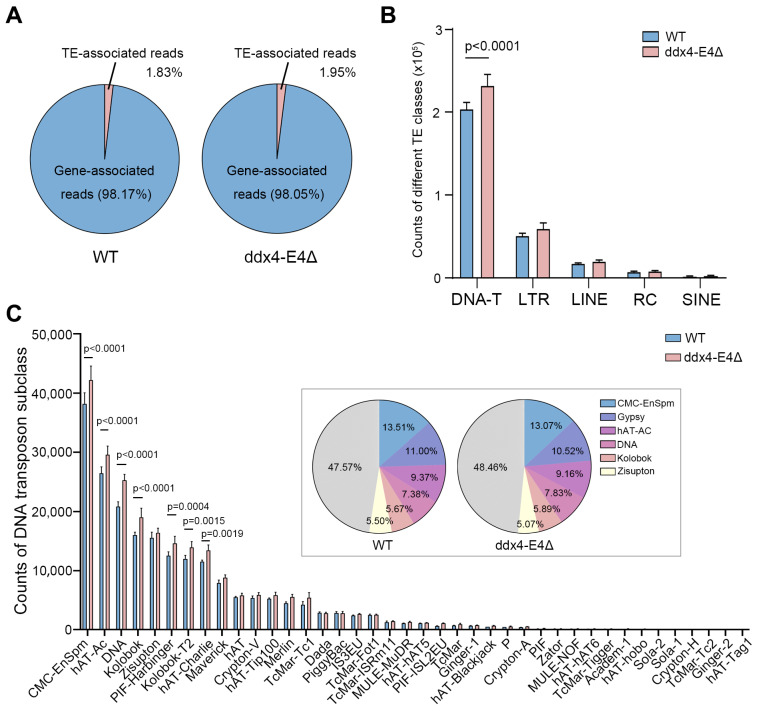
Identification of TEs and quantitative analysis of TE expression in WT and ddx4-E4Δ zebrafish ovaries. (**A**) The percentages of RNA-seq reads belonging to TEs in WT and ddx4-E4Δ ovaries. (**B**) Count numbers of different subcategories of TEs identified in WT and ddx4-E4Δ ovaries by RNA-seq. (**C**) Count numbers of DNA transposon subclasses in WT and ddx4-E4Δ ovaries.

Ddx4 protein has been reported to play a key role in piRNA biogenesis and piRNA-mediated transposable element silencing in *D. melanogaster* and *M. musculus* [8,14]. Whether the ovary-biased *ddx4-L* plays a role in TE silencing in zebrafish ovaries remains unclear. Using the RNA-seq data of WT and *ddx4-L* mutant ovaries, we identified the TEs in zebrafish ovaries and quantified their expression levels. We found that 1.83% of the reads in WT ovaries and 1.95% of the reads in *ddx4-L* mutant ovaries belonged to TEs (Figure 5A and Table S6). The activity level of TEs increased by 6.5% in *ddx4-L* mutant ovaries. Furthermore, we classified TEs into five subcategories, DNA transposons (DNA-Ts), LTR retrotransposons (LTRs), LINEs (Long Interspersed Nuclear Elements), RC (Rolling Circle) transposons, and SINEs (Short Interspersed Nuclear Elements), and analyzed their expression levels, respectively. Interestingly, only the DNA transposons were significantly activated in *ddx4-L* mutant ovaries (Figure 5B and Table S6). A more detailed analysis showed that the major subclasses of DNA-T, such as CMC-EnSpm (CACTA transposons), hAT-AC (hobo-Activator-Tam3), DNA, and Kolobok (Kolobok Superfamily Transposons), were highly expressed in *ddx4-L* mutant ovaries compared to WT ovaries (Figure 5C and Table S6). These results suggest that *ddx4-L* plays a specific role in the suppression of DNA transposons in zebrafish ovaries.

### 3.6. Identification of Differential Alternative Splicing Events (DASEs) Between WT and ddx4-L Knockout Zebrafish Ovaries

**Figure 6:**
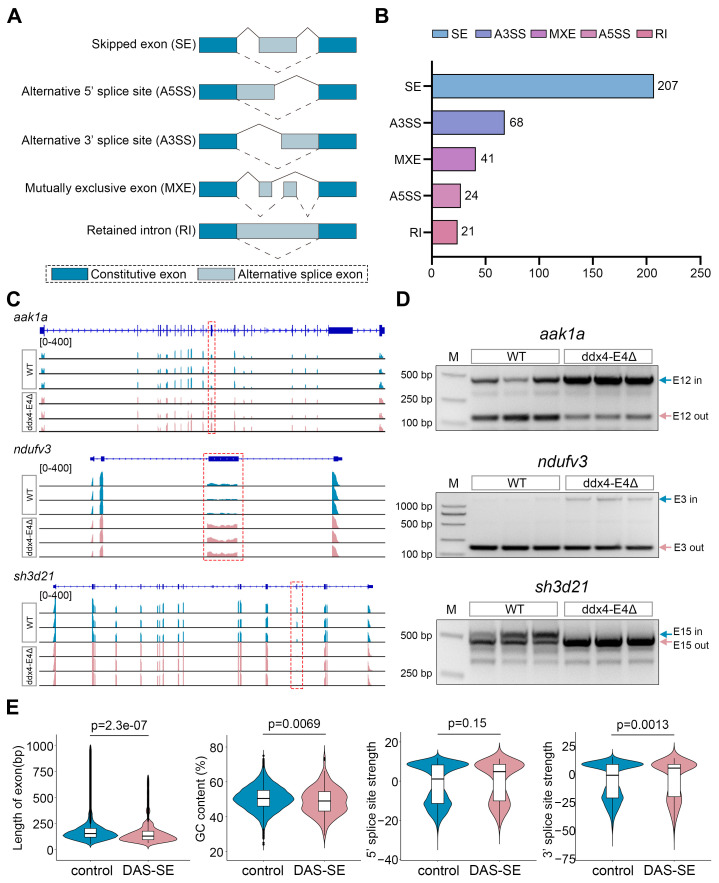
Identification of differential alternative splicing events between WT and ddx4-E4Δ zebrafish ovaries. (**A**) Five types of alternative splicing events analyzed in this study are shown. (**B**) The numbers of DASEs for each AS type between WT and ddx4-E4Δ ovaries are shown. (**C**) RNA-seq tracks of *aak1a*, *ndufv3*, and *sh3d21* are shown. The alternative exons are indicated with boxes. (**D**) RT-PCR validation of the three selected DASEs. Original gel images are shown in Figure S1B. (**E**) The differentially spliced exons affected by *ddx4-L* knockout have relatively shorter length, higher GC contents, similar 5′ splicing sites, and stronger 3′ splicing sites.

Alternative splicing of pre-mRNAs has been reported to play important roles in sex determination and gonad development [32,33]. Considering that Ddx4 is an RNA-binding protein, we wondered whether deletion of *ddx4-L* affects the alternative splicing of genes in ovaries. Five major types of alternative splicing events (Figure 6A), including skipped exons (SEs), alternative 5′ splice sites (A5′SSs), alternative 3′ splice sites (A3′SSs), mutually exclusive exons (MXEs), and retained introns (RIs), were identified by rMATs based on the RNA-seq data. A total of 361 differential alternative splicing events (DASEs) (inclusion level change ≥ 0.1 and adjusted *p*-value ≤ 0.05) were identified between the WT and *ddx4-L* mutant ovaries (Figure 6B and Table S7). SE and A3′SS were the two most abundant types of DASEs, accounting for 57.3% (207/361) and 18.8% (68/361). To validate the DASEs identified by RNA-seq analysis, we performed RT-PCR assays to detect the alternative splicing events in *aak1a*, *ndufv3*, and *sh3d21* genes in WT and *ddx4-L* mutant ovaries at 6 mpf. The RT-PCR results were highly consistent with the RNA-seq results (Figure 6C,D). Since the number of SE events was much higher than the other types, we focused on analyzing the sequence characteristics of SE events, including exon length, GC content, 5′ splice site strength, and 3′ splice site strength of the affected exons (Figure 6E). The results showed that the shorter exon length, the lower GC content, and the higher 3′ splice site strength were associated with the differential alternative splicing–skipping exon (DAS-SE) events (Figure 6E).

### 3.7. Identification of the APA Events Between WT and ddx4-L Knockout Zebrafish Ovaries

**Figure 7:**
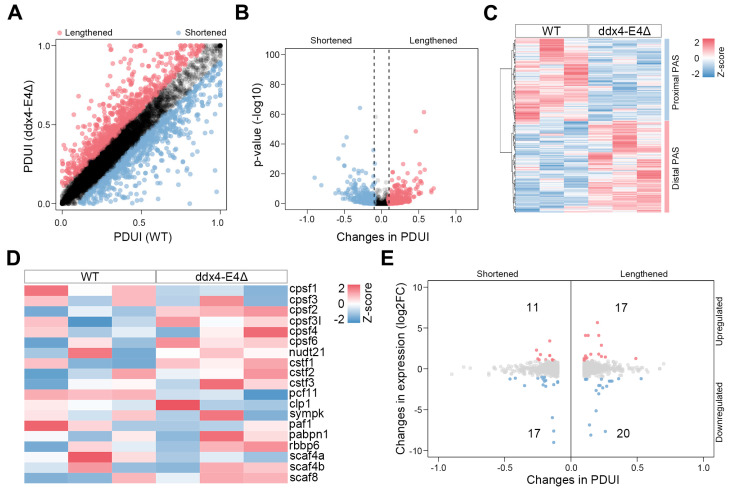
Identification of APA events between WT and ddx4-E4Δ zebrafish ovaries. (**A**) A plot of PDUI score of each gene in WT and ddx4-E4Δ groups. Grey dots indicate APA events showing no difference between WT and ddx4-E4Δ ovaries. (**B**) A volcano plot denoting 3′UTR-shorterned (blue) and -lengthened (red) gene hits. Grey dots indicate APA events showing no difference between WT and ddx4-E4Δ ovaries. (**C**) Heatmap of proximal and distal PAS usage. (**D**) Expression profile of key APA factors. (**E**) Log-fold change in gene expression is plotted against ΔPDUI for 3′UTR-altered genes. Grey dots indicate differential APA events that do not affect gene expression.

Alternative polyadenylation can mediate the dynamic use of the 3′ untranslated region (3′UTR) to regulate mRNA abundance, stability, localization, and translation at post-transcriptional level [34,35]. Based on the RNA-seq data of WT and *ddx4-L* mutant ovaries, we analyzed the APA events in adult zebrafish ovaries by using the DaPars algorithm. In total, 269 genes with 3′ UTR lengthening events (ΔPDUI ≥ 0.1, adjusted *p*-value < 0.05) and 278 genes with 3′ UTR shortening events (ΔPDUI ≤ −0.1, adjusted *p*-value < 0.05) were identified between WT and *ddx4-L* mutant ovaries (Figure 7A,B and Table S8). The differential APA events were shown in the heatmap (Figure 7C). We also analyzed the expression patterns of APA regulatory factors in WT and *ddx4-L* mutant ovaries (Figure 7E). No significant difference was observed. Furthermore, to determine whether APA events affect the mRNA levels of the corresponding genes, we performed an integrated analysis of APA events and DEGs by plotting the ΔPDUI values and the log2FC values together (Figure 7E). In 3′ UTR shortened genes, 11 genes were significantly upregulated, and 17 genes were significantly downregulated. Meanwhile, in 3′ UTR lengthened genes, 17 were significantly upregulated, and 20 genes were significantly downregulated.

### 3.8. Identification of RNA Editing Events Between WT and ddx4-L Knockout Zebrafish Ovaries

**Figure 8:**
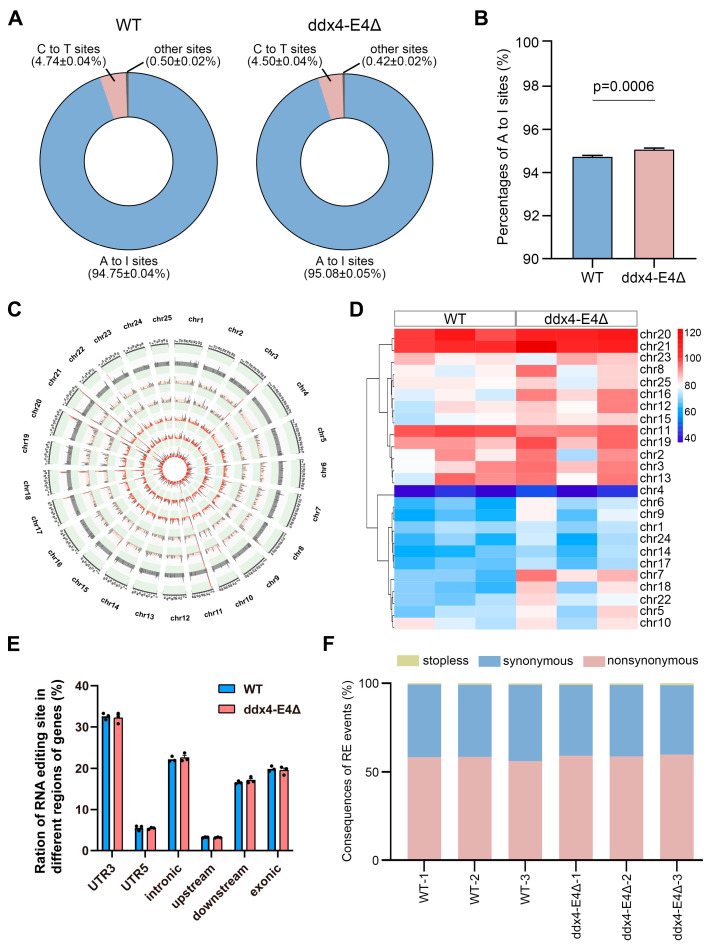
Identification of RNA editing events in WT and ddx4-E4Δ zebrafish ovaries. (**A**) Percentages of A-to-I, C-to-T, and other types of RNA editing events. (**B**) Proportion of A-to-I RNA editing events. (**C**) Distribution of RNA editing events on each chromosome. The RNA editing levels are shown with red bars. (**D**) The number of RNA editing sites on unite chromosome length of 25 chromosomes. (**E**) Distribution of the RNA editing sites in different regions of genes in WT and ddx4-E4Δ ovaries. (**F**) Functional consequences of the RNA editing sites located in CDSs.

RNA editing is a unique type of RNA modifications that may not only affect the cellular fate of RNAs but also change the protein sequences encoded by modified RNAs [36]. DEAD-box family RNA helicases, such as DDX6 and DHX9, have been reported to play regulatory roles in RNA editing [37,38]. Ddx4 is also an ATP-dependent RNA helicase of the DEAD-box family, and we wondered whether deletion of *ddx4-L* affects RNA editing events in zebrafish ovaries. Based on the RNA-seq data of WT and *ddx4-L* mutant ovaries, we analyzed the RNA editing events in zebrafish ovaries. A-to-I RNA editing was the most abundant, accounting for 94.75 ± 0.04% and 95.08 ± 0.05% of RNA editing events in WT and *ddx4-L* mutant ovaries, respectively (Figure 8A and Table S9). The *ddx4-L* mutant ovaries had a higher proportion of A-to-I events than that of WT ovaries (Figure 8B). The distribution of RNA editing sites on the 25 chromosomes were also analyzed in WT and *ddx4-L* mutant ovaries (Figure 8C,D). Compared to WT ovaries, more RNA editing events were found to be located at chromosome 7 in *ddx4-L* mutant ovaries. Furthermore, we found that a majority of RNA editing events were located in the 3′UTR and intronic regions of genes, and no region-specific differences were observed between WT and *ddx4-L* mutant ovaries (Figure 8E and Table S9). For those RNA editing events located in CDSs, approximately half of them resulted in amino acid changes (Figure 8F and Table S9).

## 4. Discussion

The sex-specific alternative splicing of exon 4 in zebrafish *ddx4* gene was identified to generate ovary-biased and testis-biased *ddx4* isoforms by RT-PCR previously [20] and transcriptome analysis recently [21]. The sex-specific functions of *ddx4* in zebrafish ovaries and testes remain largely unclear. In this study, we have made important progress in the ovarian phenotype of *ddx4-L* mutants and the underlying molecular mechanisms. We found that the abnormal differentiation of oocytes results in enlarged abdomen, immature oocyte accumulation, reduced egg production, and low fertilization rate in the *ddx4-L* mutant females. Furthermore, through RNA-seq analysis, we found that *ddx4-L* plays important roles in *sycp1* expression and DNA transposon repression, which may explain the ovarian defect of the *ddx4-L* mutants.

Complete deletion of *ddx4* in zebrafish results in all-male infertile offspring [6], which does not allow for investigation of the functions of *ddx4* in adult ovaries. However, due to the presence of the *ddx4-S* transcript, the *ddx4-L* deleted zebrafish reported here is fertile, although the number and quality of the laid eggs are both decreased. Our results suggest that the *ddx4-S* transcript can partially compensate for the functions of *ddx4-L* in zebrafish reproduction and also provide an opportunity to explore the sex- or transcript-specific roles of *ddx4* in the ovary. Interestingly, the arrest of meiotic progression has also been mentioned in the juvenile gonads of the *ddx4* complete knockout zebrafish and *Drosophila melanogaster* [3,6], indicating that *ddx4* may play a conserved role in the regulation of meiosis.

Meiosis is an essential step in generating sperm and eggs for sexually reproducing organisms, and meiotic defects can lead to infertility or decreased gamete quality [39,40]. Through RNA-seq analysis and qPCR and Western blot validation, this study, for the first time, revealed that *ddx4-L* regulates the expression of *sycp1* and *sycp3*, which are the major components of the synaptonemal complex and are required for the repair of double-strand breaks into crossovers during the meiotic process [41,42]. Knockout of *Sycp1* can lead to meiotic defects and, finally, male infertility in zebrafish and mice [25,43]. The dramatical downregulation of *sycp1* may be responsible for the abnormal accumulation of immature eggs in *ddx4-L* mutant ovaries, which is a sign of oogenesis arrest. At present, how *ddx4* specifically affects the mRNA levels of *sycp1* in zebrafish oocytes remains unclear. Considering that Ddx4 is an RNA-binding protein, validating the interaction between Ddx4 protein and *sycp1* mRNA and identifying the specific interacting region may further elucidate the underlying regulatory mechanism at the molecular level.

Egg quality is a complex biological characteristic that determines the reproductive capacity of females. In this study, through detailed reproductive phenotype analysis and ovarian gene expression profiling, we demonstrated that egg quality was significantly reduced in *ddx4-L* mutants compared to WT controls, as reflected by the low fertilization rate, the disrupted expression of genes related to fertilization regulation and sperm–egg recognition, and the upregulation of TEs. Derepression of TEs has been demonstrated to have a deleterious impact on egg quality by inducing genomic instability [18,44]. TEs can be categorized into two major classes according to their mobilization mechanisms: Class I retrotransposons (such as LTR, LINE, and SINE); and Class II DNA transposons and rolling-circle elements [45]. Previous studies have shown that Ddx4 plays an important role in piRNA-mediated transposon silencing during gametogenesis in mice and fruit flies [8,14]. Interestingly, we found that deletion of *ddx4-L* in zebrafish specifically resulted in the upregulation of only DNA transposon RNAs. How the ovary-biased isoform of *ddx4* exerts its inhibitory effect on DNA transposons remains unknown. The process of piRNA biogenesis may be involved. Otherwise, Ddx4 may directly bind transposon RNAs and repress their activities. Further studies are needed to elucidate the precise mechanism.

## 5. Conclusions

In this study, we examined the ovarian phenotype of the *ddx4-L* mutants and identified the underlying molecular mechanisms through transcriptome analysis. An ovarian phenotype of enlarged abdomen, immature oocyte accumulation, reduced egg production, and low fertilization rate was observed. Differentially expressed genes and TEs, as well as the events of alternative splicing, alternative polyadenylation, and RNA editing, were analyzed. Notably, we found that the significant downregulation of the meiotic gene *sycp1* and the derepression of DNA transposons may be responsible for the ovarian defect of *ddx4-L* mutants. Our study revealed novel sex-specific functions of *ddx4* in regulating oocyte meiosis and DNA transposon repression.

## Data Availability

The data presented in this study are available in the Materials and Methods section and in the Supplementary Materials section. The RNA-seq data are available under the GEO accession code GSE276129 (https://www.ncbi.nlm.nih.gov/geo/, accessed on 31 August 2024).

## Supplementary Materials

The following supporting information can be downloaded at https://www.mdpi.com/article/10.3390/biology13121055/s1, Table S1: List of primers used in this study; Table S2: Quantitative data of gonad weight, GSI, fertilization rate, and number of eggs produced of WT and *ddx4-L* mutant females; Table S3: RNA-seq data quality control; Table S4: Differentially expressed genes between ovaries of WT and *ddx4-L* mutants; Table S5: GO and GSEA enrichment analyses for DEGs; Table S6: Identification and expression analysis of TEs in WT and *ddx4-L* mutant ovaries; Table S7: Differential AS events between WT and *ddx4-L* mutant ovaries; Table S8: APA analysis in WT and *ddx4-L* mutant ovaries; Table S9: RNA editing analysis in WT and *ddx4-L* mutant ovaries. Figure S1: Original images for Figure 4C and Figure 6D.
