# Supplementary figures and images for: Deletion of ddx4 Ovary-Specific Transcript Causes Dysfunction of Meiosis and Derepress of DNA Transposons in Zebrafish Ovaries
Source: Biology (Basel). 2024 Dec 16;13(12):1055. doi: 10.3390/biology13121055 (PMC11673608; doi:10.3390/biology13121055)

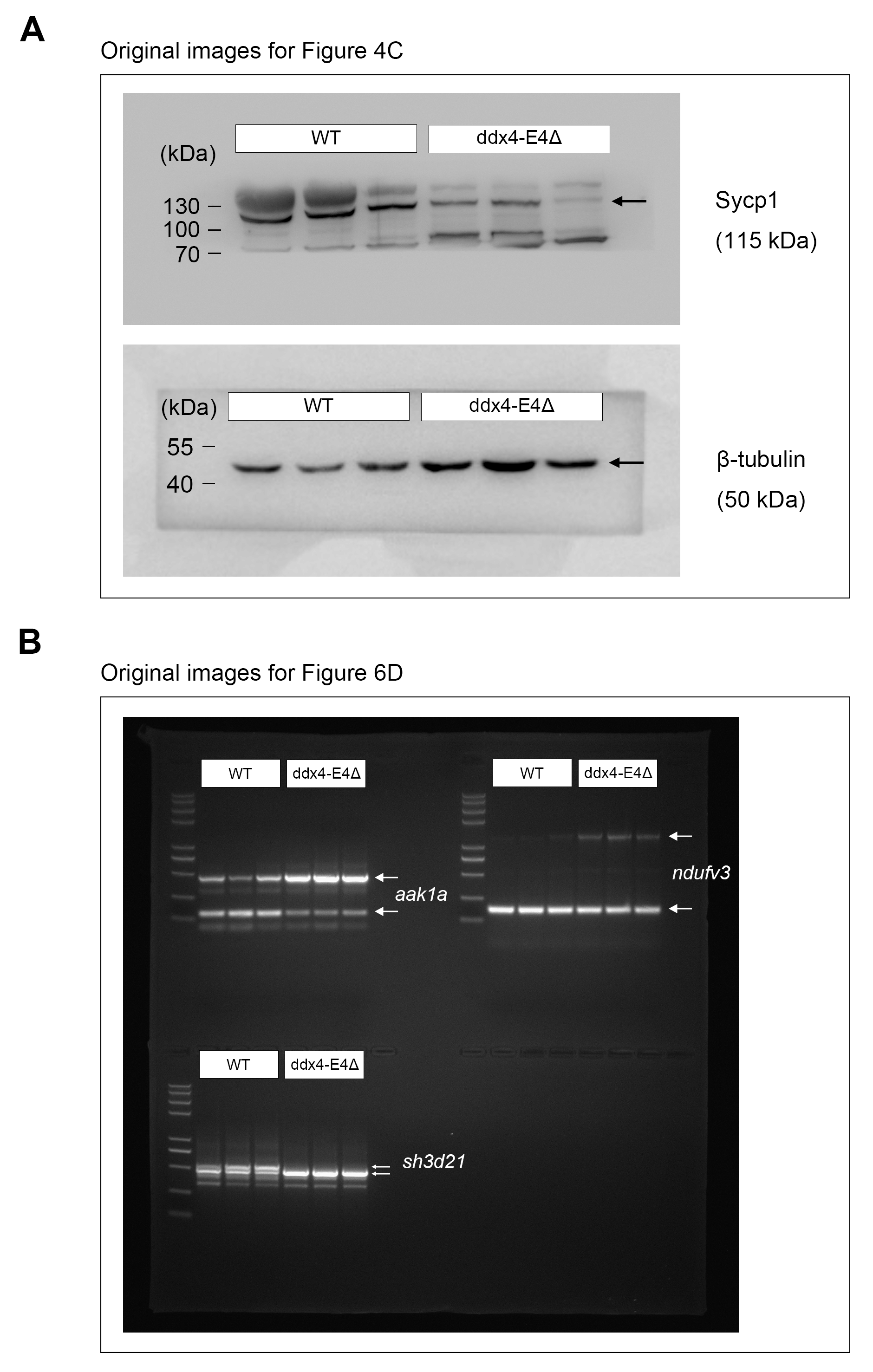

Supplement: Supplementary file 1 [file biology-13-01055-s001.zip › Figure S1 R1.tif]
